# Lipid metabolism on and off the beaten path

**DOI:** 10.1016/j.jbc.2025.110697

**Published:** 2025-09-10

**Authors:** Rosalind A. Coleman

**Affiliations:** Departments of Nutrition and Pediatrics, University of North Carolina at Chapel Hill, Chapel Hill, North Carolina, USA

I had always longed to do something special with my life, but for a long time, I was unsure about what it might be. As a teenager, I was interested in nature and novels, in drama, history, and painting. My mother, always hopeful for expert insight, took me to a career counselor. After a multitude of personality and aptitude tests, he opined that I would do well as an occupational therapist. I was horribly disappointed, but in fairness to him, few women in the 1950s embarked on scientific careers.

Ours was a striving family, free of the constraints of my grandparents’ immigrant background. My father, the only one in his family to get beyond high school, was a dentist and my mother ran community fundraisers for the parent-teacher association and, in middle age, became a stockbroker. We enjoyed the ease of suburban life in a small town—walking to school, gardening, swimming in the sea-weedy Long Island Sound, drawing and painting, playing baseball—and on Sundays, we visited relatives in Brooklyn, or they came to us. My parents prized education and what they considered high culture—Brahms and Mozart, Rembrandt and Picasso, “good” literature. My two brothers and I were each encouraged to play an instrument (piano for me), and we were taken to art museums and concerts in New York. Unlike the career counselor, I did not imagine that any profession would be closed to me or that I would be restricted in any future endeavor.

Off I went to Harvard (Radcliffe College), having decided to major in psychology since the only woman I knew who had a job other than teaching was a child psychologist. But I soon dropped my psychology major. I disliked the uncertainty, the absence of a predictable outcome. Of course, I would come to learn that medical practice and scientific research have few certainties, but it did not seem that way to me at that time. I believed that diagnoses were definitive, that cures were always possible.

I also dropped my second major, political science, which forced us to memorize theories of government and the unstable structures of French and Soviet bureaucracies—extremely boring, I thought, but what then? Finally, after a year of George Wald’s brilliant lectures (before he had won his Nobel Prize for discovering the mechanism of vision), I changed course for the third time and decided to major in biology.

It turned out to be lucky for me that my high school science courses had lacked information about anything more recent than the structures of plant stems and the workings of pistons and levers. I had never seen an electron micrograph and was unaware that the insides of cells were crowded with twisting membranes and complex organelles. I had never seen the gorgeous structure of vitamin B12 with its intricate chemical rings and its central molecule of cobalt. Perhaps I imagined a deep shimmering blue, like the oil paint color. I was mesmerized. Here at last was a field of study that seemed endlessly fascinating, that centered on the meaning of life and had truth at its essential core.

Despite my enthusiasm, I was uncertain about how to begin a scientific career. The members of Harvard’s science faculty were intimidating and unencouraging. Nearly all were famous men (no women, of course) who personally knew the major scientists whose groundbreaking ideas and experiments we were studying. Several were themselves celebrated for inspired work that had altered major fields. Some even had experiments named after them. Thus, I was taught by Matthew Meselson of the Meselson–Stahl experiment, who proved that each new strand of DNA took as its template one of the former strands of the DNA duplex, and by Jim Watson, who had just received the Nobel Prize for his work with Crick on DNA structure.

Learning laboratory techniques came to me readily. My laboratory instructor commended me for being the only one of thirty students in my section to successfully generate penicillin-resistant *Escherichia coli* bacteria through plasmid transformation. I also excelled at a summer job studying the effect of thiamine deficiency on pyruvate dehydrogenase activity in rat brain homogenates. Despite my technical accuracy using lambda mouth-pipettes, Pierre Dreyfus, my summer laboratory director, never suggested that I might have a future in science. Even more disappointingly, my course directors actively discouraged me from pursuing research. I felt ignored and adrift.

Because it was clear to me that no one believed I should go to graduate school, my fallback was medicine. Although my family rarely mentioned antisemitism, and I never experienced it firsthand, medicine was held up as a profession in which you were your own boss and could not be fired (with the obvious implication that other professions would be less secure). So, medicine had been placed almost unconsciously in my mind as a profession particularly suited to a stigmatized group like Jews or women. Despite us never seeing a single female professor, the Radcliffe president assured us that we could be anything we wanted. When I sought advice from the Dean of Students, a large bosomy woman who believed that the majesty of a Harvard degree would overcome any possible deficiency, she assured me, “You’ll be accepted anywhere you apply, dear.”

After a 3-h conversation (more of a debate, actually), I convinced my advisor, Peter Albersheim, to write a strong recommendation letter, but he, like my other science professors, questioned my decision, even betting me a dinner that I would drop out of medical school because that was what women did. (Years later, I waylaid him at a science conference in DC, told him that he had lost the bet, and demanded that he at least take me out to lunch.)

In the 1960s, women comprised a mere seven percent of the total first-year US medical school class of almost 8600. Some schools admitted only one woman per class or none at all. Before my interviews, I practiced answering the questions that I knew the interviewers were likely to ask me, questions that we think of as inconceivable today, such as “We have such a limited number of places in our class— why should we accept you when you’ll just get married and drop out?” or “How depressed are you on “those” days of the month?” Women applying to medical school expected to be probed by such questions; we recognized that female doctors were anomalies and that places in medical schools were reserved for those more likely to succeed, namely men.

## Medical school and internship

In any case, I was accepted by two of the six schools to which I had applied and chose Case Western Reserve School of Medicine in Cleveland. I was one of only eight women in my class of 80, but I felt supported and encouraged by the faculty and my fellow students, and I spent 4 years studying very hard and protesting against the war in Vietnam. Always short of money, I volunteered for several ill-conceived clinical “studies.” Among them, I learned what it feels like to have a blood glucose of 20 mg/dl, (It felt like an out-of-body experience where I floated above the hospital room with the heart-pounding certainty that I would die), and what a blood glucose of 1200 mg/dl feels like (I felt woozy, headachy, and disoriented, with the desperate fear that I would pee a huge quantity of urine all over the floor if they could not quickly find me a bedpan). These visceral experiences were my first intimations of insulin’s profound control of metabolism — a very physical entry into a field that would underpin my future research life.

Pediatrics seemed like a natural choice for internship. I had been impressed with the implacable commitment of the pediatric faculty members I had met, and I felt that I could establish a strong rapport with parents. Unlike adult patients whose lifestyles had harmed their own health and who ignored important advice and failed to take their meds, I saw myself and the parents both focused on doing our best for the child under our mutual care. Perhaps even more importantly, every female physician I had encountered during medical school was a pediatrician.

At New York Hospital (now Weill Cornell Medicine), our internship schedule was exhausting. On weekdays, we worked every day, every other night, and every other weekend as well—about one hundred hours a week of sleepless nights and days full of admissions, discharges, patient exams, minor and major emergencies, patient rounds, conferences, and oral reports to the private pediatricians whose patients we were taking care of. We drew blood from infants and toddlers, started all the IVs, and ran many of the laboratory tests ourselves, including bacterial cultures and blood gases for the premature infants. Like many of my fellow interns, I drugged myself with dextroamphetamine to keep going. Apollo 11 landed on the moon in July, but I can remember little else that happened outside of the hospital for most of that first year.

The second year was similarly exhausting, and when I finally paused to reflect during my 2-week vacation, I decided to stop mindlessly trudging on to the next thing. I canceled my interviews for a neurology fellowship and left the residency program to bum around South America. I had no clear plan and contemplated the possibility of never returning to medicine.

## New directions in North Carolina

Travel gave me time to think and a renewed sense of commitment to medicine. At the end of almost a year of hitchhiking from Bogota to Punta Arenas to Rio de Janeiro, I flew to Durham to visit a friend from Cleveland who was completing her postdoc in Chemistry at Duke. After a hastily arranged interview at which I wore the few clothes in my backpack that were unripped, unstained, and relatively clean, I was offered a job at Duke as a Pediatric Senior Resident. With this job, less sleep-deprived and more enjoyable than my first, I gravitated toward the detective work of diagnosing children with esoteric genetic diseases, mostly involving mutations in carbohydrate and amino acid metabolism. By the end of the year, I had decided to pursue a fellowship in metabolic disease.

But before I began my fellowship, I was offered another opportunity to travel, this time overland from France to India and Nepal. One last try, I thought, to be certain that medicine was the right future. I deferred my fellowship, flew to France, and then hitchhiked through Turkey, Iran, Afghanistan, and Pakistan, a trip that would be impossibly dangerous today. The overland trail from Europe to Nepal was populated with young people, each on some interior journey toward self-transformation. I halfheartedly searched for a medical job in Kathmandu but managed to avoid yet another meaningless diversion. Back in Delhi, I paid cash for a ticket from a back-alley scalper, boarded a Syrian Airlines flight to London, and returned to Durham. Drifting without purpose had become unsatisfying; I was ready to commit.

## Turning toward research

**Figure 1:**
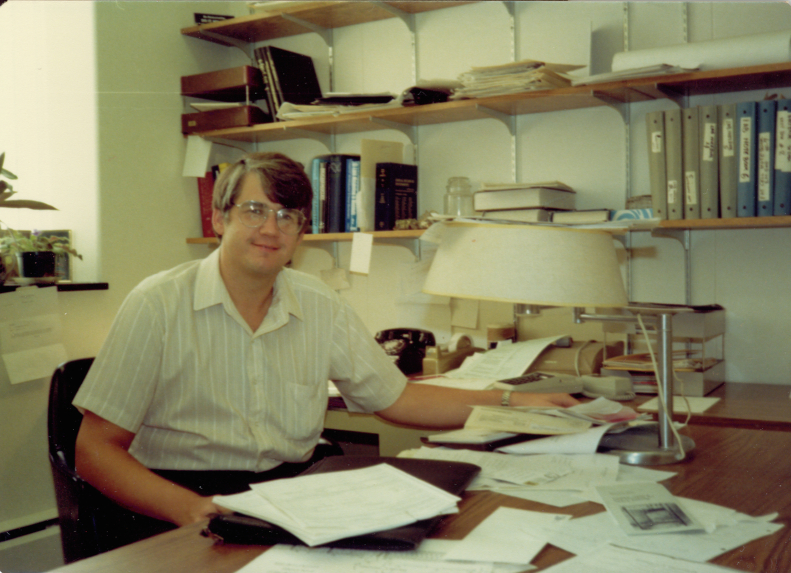
**Robert M. Bell at his desk**.

It was Jim Sidbury, Chief of Duke’s Pediatric Metabolism Division, who renewed my interest in basic science. Because, he said, I would never have enough patients with genetic metabolic diseases to justify an academic salary, I would need to do research. He sent me to interviews with four faculty members in the Department of Biochemistry, and because I was impressed by his enthusiasm and his calm demeanor, I chose Robert (Bob) M. Bell’s laboratory, both of us fully aware that I knew nothing about research (Fig. 1).

Bob hoped I would investigate phospholipid synthesis in *E. coli*, but I insisted on working on a problem that seemed more clinically relevant and so began my entry into the world of triacylglycerol (TAG), adipocytes, diabetes, and fatty liver. My first project, using rat adipose tissue, characterized the final enzyme in the pathway of TAG synthesis, diacylglycerol acyltransferase. That characterization, my first paper, was published by the *Journal of Biological Chemistry* (JBC) (1). Looking back nearly 50 years later, the errors in our interpretation are obvious; I had thought that I was describing a single enzyme, but we did not know then that every one of the steps involved in the synthesis of TAG and the major phospholipids was catalyzed by two or more independent enzymes, each the product of a separate gene.

My second project, also published in the JBC, asked whether phosphatidylcholine and phosphatidylethanolamine were synthesized by one or two independent enzymes (2). Again, the current view, based on molecular cloning and mouse knockouts, is considerably more complicated than we had imagined at that time.

**Figure 2:**
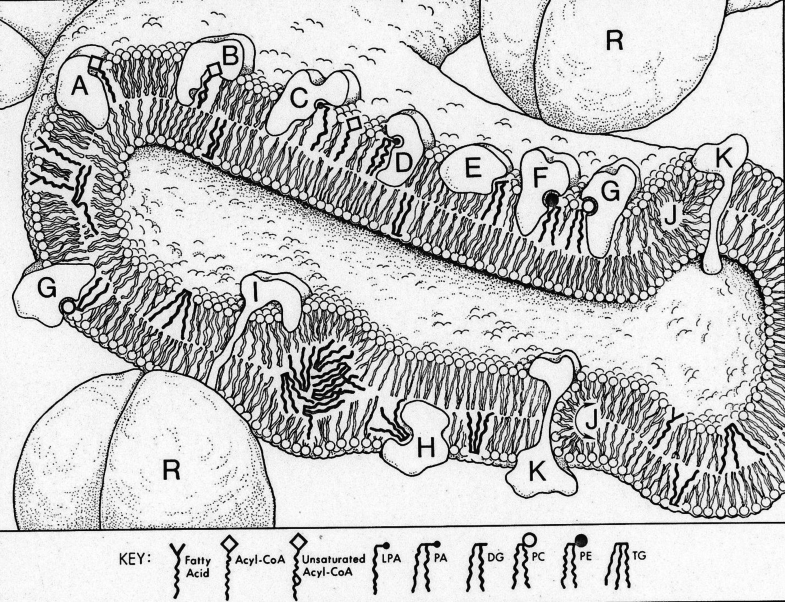
**Topography of enzymes of glycerolipid synthesis in the ER bilayer (circa 1980).***A*, acyl-CoA synthetase; *B*, fatty acid desaturase; *C*, glycerol-3-P acyltransferase; *D*, lysophosphatidic acyltransferase; *E*, phosphatidic acid phosphatase; *F*, diacylglycerol ethanolaminephosphotransferase; *G*, diacylglycerol cholinephosphotransferase; *H*, diacylglycerol acyltransferase; *I*, apoB; *J* and *K*, phospholipid flippase and mechanism; R. ribosome. ER, endoplasmic reticulum.

In 1978, the asymmetric positioning of phospholipids on each side of membrane bilayers had been documented, but no one understood how this asymmetry might occur. Thus, my next and most demanding project was to determine whether the synthesis of TAG, phosphatidylcholine, and phosphatidylethanolamine took place on the outer or the inner aspect of the endoplasmic reticulum (ER) or, perhaps occurred in differing amounts on each side of the ER to match the composition of the two monolayers. Our article, showing that the active sites of seven of these ER enzymes faced the cytosol, implied that the formation of membrane bilayers requires phospholipids to flip in defined percentages from one side of the ER bilayer to the other and that the construction of other cell membranes demands that newly synthesized phospholipids must be transported to them (3). Interestingly, although we were aware that each of the enzymes is an intrinsic membrane protein, none of them span the membrane bilayer in our drawing (Fig. 2).

These membrane topography experiments sparked my interest in the structural nature of cells—how they are organized internally so that enzyme pathways can be regulated independently, and how each member of a pathway hands off its product to a specific subsequent enzyme, somewhat like the way a baton is handed off during a relay race.

We were shocked and disappointed when the JBC rejected our topography paper. I learned later that, despite the fact that the primary reviewer could not find anything wrong with our methods or our interpretation, he just could not believe our results. In any case, we published in the *Journal of Cell Biology* (3). This was not the last time I encountered a scientist unable to reconsider his own long-held belief. Some years later, I proposed to determine how fatty acids were transported across the placenta to the developing fetus. One of the scientists on the National Institutes of Health (NIH) review panel rejected my proposal because he “knew” that the placenta did not transport fatty acids at all, even though the fetal brain is replete with omega-3 and omega-6 fatty acids, which cannot be synthesized by mammals and must originate in the maternal diet.

At the 1977 FASEB conference, I heard Daniel Lane from Johns Hopkins describe the increases in the activities of enzymes of *de novo* fatty acid synthesis in 3T3-L1 cells as they differentiated into adipocytes. I introduced myself and convinced him that we should examine the enzymes of TAG synthesis in these cells. This proved to be a wonderful collaboration. We had no tissue culture facility at Duke, so Dan’s lab collected the cell membranes and shipped them to North Carolina where I measured enzyme activities. Because a differentiation cocktail had not yet been invented, we had to allow the cells to differentiate spontaneously. To ensure that we would be able to study the cells at their moment of differentiation, Dan’s postdoc, Brent Reed, collected cells every other day for 2 weeks. I found that the enzyme activities in the TAG synthetic pathway increased simultaneously and dramatically, with the activity of long-chain acyl-CoA synthetase (ACSL; formerly known as fatty acid CoA-ligase) increasing 100-fold (4, 5). I would never have guessed that I would rely on this information 30 years later when we created a mouse deficient in adipose ACSL1 (6).

**Figure 3:**
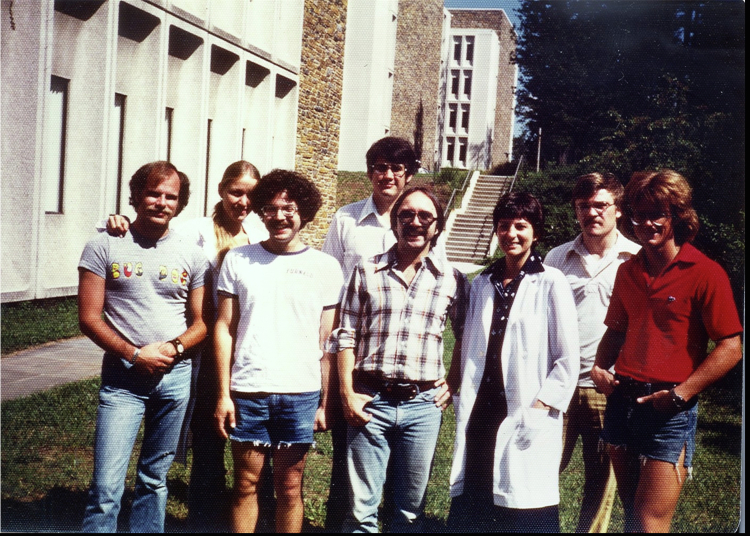
**Members of Bob Bell’s lab in 1976.** Thomas McIntyre, Virginia (Val) Lightner, David Schlossman, Bob Bell, Mark Polokoff, RAC, Casey Jason, and Ralph Edgar outside the Nanaline Duke building.

**Figure 4:**
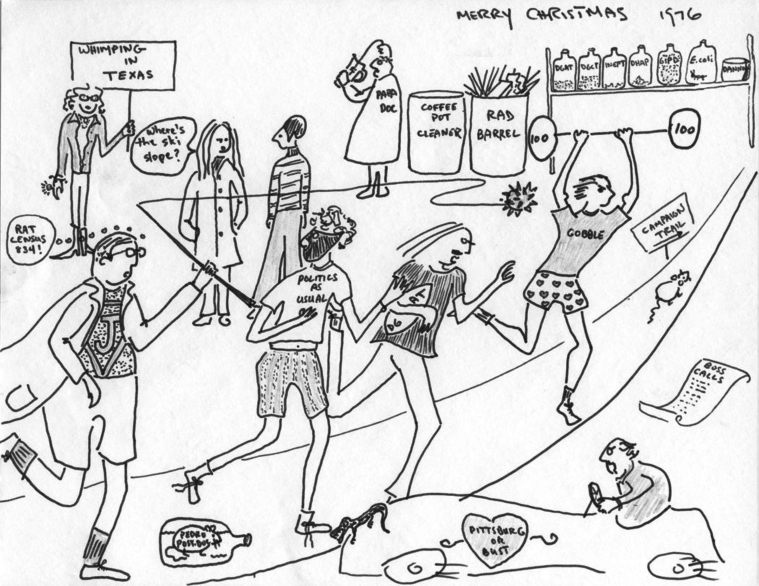
**Christmas Cartoon 1976**. Bob (“J” for Jefe) bemoans our expensive rat census, Tom McIntyre leaves for his postdoc in Pittsburg, and the graduate students, technicians and equipment embody laboratory jokes and jargon. (I am the person in bell bottoms and striped shirt.)

**Figure 5:**
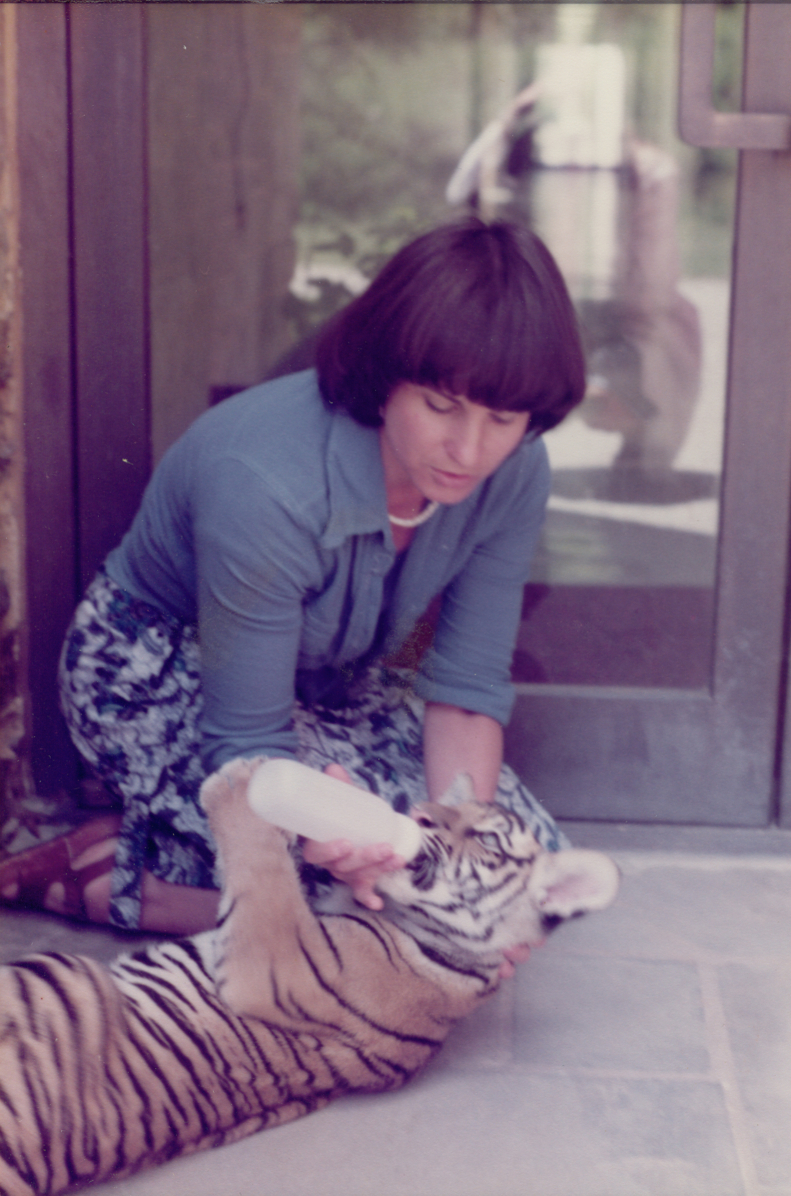
I bottle-feed a baby tiger in the Nanaline Duke building.

I loved working in Bob’s laboratory. Unlike the stresses of clinical work, I loved planning experiments, assaying enzymes, and plotting graphs. Bob’s students were fun to talk to, and we had great discussions that ranged from phospholipid synthesis to politics to Duke basketball (Fig. 3). I drew a picture for each departing student as well as a yearly Christmas cartoon for the laboratory (Fig. 4). Every year the Biochemistry Department had a huge Thanksgiving meal in the main hallway, replete with turkeys and pies cooked in our laboratory ovens (!), and the younger faculty members joined students for dancing and dress-up Halloween parties. The departmental atmosphere was generally relaxed: Some of us jogged together, we sampled home-made beer in our laboratory, and I learned to hit golf balls, although not very well. Bob’s students often played bridge in an unused room, and one of Paul Modrich’s technicians who had volunteered to socialize a baby tiger, often brought it to work (Fig. 5).

When my fellowship ended, Duke offered me a position as Assistant Professor of Pediatric Metabolism. I accepted, believing that my responsibilities included taking care of patients who had genetic diseases and establishing my own research laboratory. This offer was not a good one. I was paid minimally more than my postdoc salary, and the man heading my specialty division said that he expected me to supervise his laboratory. My refusal to accept this role led, unsurprisingly, to a hostile environment. It was a poor beginning, but even worse, because I had never heard of the concept of “start-up funds,” I did not ask for any. I believed that I was totally on my own to find enough money to support my research. I assumed that all my research money had to come from grants, so I sat down to write.

My application for a March of Dimes Basil O’Connor award necessitated a personal interview, which took place in a hotel room at a Key Largo resort. The sunburned vacationing interviewer spent most of our time together on the telephone, arguing with a colleague in New York, and terminating his conversation by saying, “I have got a beautiful girl here who wants her money.” In any case, my proposal was successful, and it allowed me to hire a technician, the wonderfully efficient and insightful Elaine Haynes (subsequently Bardes).

My NIH proposal was also funded on the first try. I had proposed to use Christian (Chris) Raetz’s filter paper disc method to discover novel cellular lipases in CHO cells. Elaine and I sorted through hundreds of discs in a search for lipase mutants, but our experiments were literally a mess. The cell colonies merged together, the intensity of the red dye stain for undegraded TAG was inconsistent, and the colonies that we selected did not regrow. Not a single thing that I had proposed in my research plan was successful.

Because the lipase project was a complete failure, I proposed a different project for my NIH renewal. This project was based on a handful of experiments that showed a dramatic increase in the activities of TAG-synthesizing enzymes in rat liver just after birth. I hypothesized that the pups suckling on a high fat milk diet would be enabled by these changes to resynthesize TAG for secretion in very low density lipoprotein. The proposal was not funded, but my chairman grudgingly found $5000 of bridge money for me, and I managed to score another $4000 from an internal Duke reproductive health grant. Elaine and I borrowed chemicals from other laboratories, scavenged equipment parts and office supplies from Duke’s surplus store, and bred our own baby rats illegally in a storage room across the hall from our lab. At the end of nine impoverished months, we published our new results (7). My resubmitted NIH proposal got a great score and remained funded for most of the following 35 years (HD19068, then DK56598).

During my 10 years as a member of the Duke pediatric faculty, I also diagnosed and cared for children who had genetic errors in metabolism. DNA genetic analysis had not been invented, so making a diagnosis demanded wide reading, symptom recognition, and detective work, all of which I enjoyed immensely. Interventions, mostly dietary, were possible for disorders such as aldolase B deficiency, medium-chain acyl-CoA dehydrogenase deficiency and certain urea cycle defects, and I participated in my astute colleague Y. T. Chen’s innovative plan to maintain euglycemia in children with glycogen storage disease (glucose-6-phosphatase deficiency). The children ate uncooked cornstarch at night, which acted as a kind of slow-release glucose source (8), and we had the pleasure of watching the children grow normally. An attempt with my good colleagues Del Wigfall and Marjorie Tripp to tackle pediatric obesity, insulin resistance, kidney disease, and atherosclerosis was prescient, but we were denied permission to establish a metabolic clinic.

I also dealt with persistent misogyny, some of it amusing. As a member of Duke’s Committee on the Status of Women, I presented data to Duke’s trustees that showed that Duke lagged behind most other medical schools in the number of its female faculty members. In response, one of the trustees drawled in a marked Southern accent, “I don’t think a lot of women are interested in blood and gore.” I like to think that my response was admirably restrained; instead of reminding him that women are used to seeing plenty of blood and gore on a monthly basis, I merely repeated the statistics. The misogyny was not unique to me. I recall enjoying a collaboration with my husband in analyzing a 1971 anatomy textbook written by three Duke professors. This text was remarkable for both its puerile and sexist writing and its use of photos of naked women in lascivious poses, purportedly to demonstrate anatomical points of interest, mostly involving breasts and buttocks (9).

But some events were painful, as when my chairman mocked my Grand Rounds lecture on cholesterol by asking, “All I want to know is what James [my husband] gets for dinner.” Even more disturbing was a clinical conference convened to discuss an infant who was born with a disorder of sexual development. When I questioned the chairman of urology about his decision to surgically “make the baby into a girl” without informing the parents, he asserted—to roars of laughter from the residents and junior faculty (all male)—that female physicians, in his view, were almost certainly surgically altered men.

Research and scientific meetings were less fraught. Although studies of adipose tissue, obesity, and TAG synthesis and storage were almost a backwater for lipid biochemists, I remained intent on understanding the connections between TAG and clinical disorders. In addition to our studies of post natal lipid metabolism (7, 10, 11), our group discovered that an alternate monoacylglycerol pathway of TAG synthesis, previously found only in the small intestine, was present in adipose tissue and neonatal liver and that the monoacylglycerol acyltransferase (MGAT) activity in the liver differed considerably from the better-known gut isoform (12, 13, 14, 15).

## A move to the University of North Carolina

My life changed radically when Steve Zeisel, the newly hired chair of Nutrition, recruited me to University of North Carolina (UNC). I am almost embarrassed to admit that with this move, my salary literally doubled—not because UNC was so bountiful, but because I had been so grossly underpaid at Duke. Steve also helped to get my grant transferred from Child Health and Human Development to Diabetes and Digestive and Kidney Diseases, a more generously funded NIH Institute. In addition to teaching a wide-ranging course on metabolism, I helped direct our Nutrition-Obesity Research Center and mentor its trainees, and I reviewed NIH and American Heart Association grants. I also organized and coorganized FASEB (Molecular Biology of Intestinal Lipid Transport and Metabolism, 2003; Lipid Droplets: Metabolic Consequences of Stored Neutral Lipids, 2007), Gordon (Molecular and Cellular Biology of Lipids, 2011), and Keystone (Type 2 Diabetes, Insulin Resistance and Metabolic Dysfunction, 2011) conferences on lipids and diabetes.

In Chapel Hill, our lab continued to study MGAT, and we used Triton X-100/phospholipid mixed micelles to understand the regulation of MGAT by membrane-associated lipid components. These studies, initiated by Ganesh Bhat, a talented associate who subsequently became a pharmaceutical consultant, showed that MGAT could be regulated by *sn*-1,2-diacylglycerol (16), sphingosine (17), anionic phospholipids (18), and monoradialglycerols (19). Ganesh was capably assisted by my technician, Ping Wang, and we all had many long discussions about whether these findings would be relevant for membranes *in vivo*.

The most beneficial part of my move to UNC was the ability to recruit and train graduate students. Because Duke had denied me access to students despite a secondary appointment in Biochemistry, I had previously relied on technicians and undergraduates to populate my laboratory.

**Figure 6:**
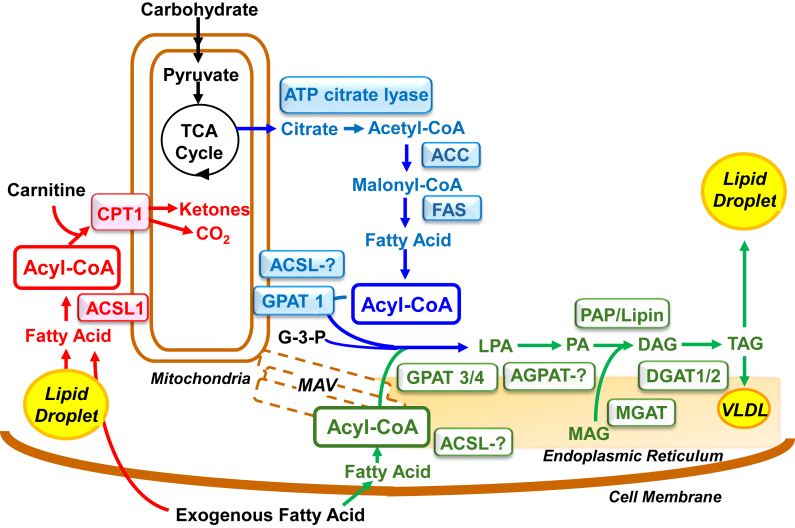
**Compartmentalization of acyl-CoAs and acyl-CoA metabolism**. Blue pathway is controlled by SREBP1 (sterol regulatory element–binding protein-1) and ChREBP (carbohydrate-responsive element-binding protein) which upregulate mRNAs for enzymes of *de novo* lipogenesis and GPAT1 to produce acyl-CoAs destined for TAG synthesis. Red pathway is controlled by PPARγ which upregulates mRNAs for ACSL1 and CPT1 to channel lipid droplet-derived fatty acids and exogenous fatty acids into the pathway of mitochondrial β-oxidation. Green pathway: fatty acids from multiple sources can be channeled into the final pathway of complex lipid synthesis. ACC, acetyl-CoA carboxylase; ACSL,long-chain acyl-CoA synthetase isoform; AGPAT, acylglycerol-3-P acyltransferase isoform; CPT1, carnitine phosphotransferase-1; DAG, diacylglycerol; DGAT, diacylglycerol acyltransferase; FAS, fatty acid synthetase; G-3-P, glycerol-3-phosphate; LPA, lysophosphatidic acid; MAG, monoacylglycerol; MGAT, monoacylglycerol acyltransferase; MAV, mitochondria-associated vesicles; PA phosphatidic acid; PAP, phosphatidic acid phosphatase; TAG, triacylglycerol; VLDL, very low density lipoprotein.

The most critical decision for any laboratory is selecting the primary research focus. I had long been interested in the relationship between glycerolipid synthesis and insulin resistance, so improved financial stability and the intellectual stimulus of postdocs and graduate students enabled me, finally, to concentrate on lipid metabolism. One of my first graduate students was Debbie Muoio—now a professor at Duke—a woman with inexhaustible energy who never willingly lost an argument. She was determined to investigate lipid metabolism in skeletal muscle. Because leptin causes weight loss in excess of a diminished intake of food and because muscle expresses leptin receptors, she predicted that leptin directly alters muscle energy metabolism. Her work demonstrated that leptin increases fatty acid oxidation in mouse soleus muscle while reducing the incorporation of fatty acids into TAG—the opposite effect of insulin (20, 21). She showed that AMP-activated protein kinase reciprocally regulates TAG synthesis and fatty acid oxidation in both hepatocytes and soleus muscle (22), likely *via* the mitochondrial glycerol-3-P acyltransferase (now GPAT1), which catalyzes the esterification of long-chain acyl-CoAs to the *sn*-1 position of glycerol 3-phosphate to form lysophosphatidic acid. Thus, when ATP levels are low, AMP-activated protein kinase-mediated inactivation of GPAT1 would enable more acyl-CoA to enter the mitochondria for oxidation. This was the first intimation that GPAT1 might regulate the incorporation of fatty acids into the pathway of TAG synthesis *versus* their entry into β-oxidation and suggested that carnitine palmitoyltransferase-1 and GPAT1 might compete for acyl-CoAs to control this partitioning (Fig. 6).

Laboratory progress was further advanced by Tal Lewin, a meticulous and thoughtful postdoc, who identified four amino acid motifs in GPAT1 that are essential for its catalytic activity (23). With her strong background in bacterial lipid biochemistry, Tal used *E. coli* for her experiments after demonstrating that these acyltransferase motifs are highly conserved across a wide variety of organisms, including other lipid acyltransferases. The motifs were located in the N-terminus region of the mitochondrial GPAT1, the region studied by Maria Gonzalez-Baró, now a Professor of Biochemistry at the University of La Plata, who had arrived from Argentina to analyze GPAT1’s structural topology and its integration into rat liver mitochondrial membranes (24). Tal’s motifs were also present in two new GPAT isoforms that we later identified: GPAT2 (25) and GPAT4 (26), the latter *via* a collaboration with Karen Reue’s group at UCLA.

These studies furthered our interest in intracellular lipid channeling. Colleagues had discovered that each step in the synthesis of TAG was catalyzed by multiple isoenzymes, and we wondered why these independent, yet structurally related, isoforms had persisted in mammals over the millennia. It seemed unlikely that their functions were merely redundant. Growing evidence, albeit indirect, suggested that cellular fatty acids might be selectively partitioned either toward degradation or toward complex lipid synthesis, depending on which enzyme catalyzed their activation to form acyl-CoAs and which catalyzed the initial esterification reaction. These pivotal steps are carried out by five long-chain ACSL and four GPAT isoforms (27, 28), so our laboratory began to investigate enzymes in both categories.

Although I had been encouraged by Eugene Kennedy’s assertion that “God didn’t make an enzyme that couldn’t be purified,” our attempts to purify MGAT were only partially successful, and we turned to cloning. Because our cloning design was based on Tal’s acyltransferase motifs, it was not altogether surprising that Ganesh Bhat cloned rat GPAT1 instead of MGAT (29). Using his sequence data and with critical help from our UNC colleague Nobuyo Maeda, we made one of the first mouse knockouts of a lipid synthetic enzyme. Linda Hammond, a serious and determined graduate student who is now a technical writer, showed that compared to WT mice, the liver from the GPAT1^−/−^ mice contained a lower content of palmitate at the *sn*-1 position of TAG, phosphatidylethanolamine, and phosphatidylcholine and a higher content of arachidonate at the *sn*-2 position of these two phospholipids (30). Because these results had been predicted by early characterization studies of GPAT1’s activity, the initial reviewers rejected our manuscript as being “only confirmatory.” This rejection ignored the novel findings of major decreases in gonadal fat, very low density lipoprotein secretion, and the content of hepatic TAG. The latter was the reverse of the almost 3-fold increase in TAG mass after GPAT was overexpressed in CHO cells (31). Taken together, these were remarkable data (recognized by *Molecular and Cellular Biology* (30)) and suggested that GPAT1 controls TAG synthesis in the liver, even though GPAT1 is located on the mitochondrial outer membrane and is not present on the ER where TAG synthesis occurs. This was yet another indication of lipid channeling.

Tal Lewin also showed discrepancies between mRNA and protein expression in different tissues. In liver and adipose, GPAT1 expression and activity decrease as much as 50% and overshoot normal levels after refeeding, suggesting hormonal and dietary control of TAG synthesis *via* GPAT1 (32). Tal and Linda Hammond then showed that GPAT1 directs exogenous fatty acid metabolism and is essential for the metabolism of excess acyl-CoAs in hepatocytes and liver (33, 34). Contributing to these concepts, Doug Mashek, a postdoctoral fellow who left studies of lipids in dairy cows to focus on cellular dynamics and fatty acid movement within cells, demonstrated that fatty acid uptake by cells is driven by downstream metabolic demand rather than by specific transport proteins (35, 36, 37). Doug is now Professor of Biochemistry, Molecular Biology, and Biophysics at the University of Minnesota, where his ironic sense of humor no doubt invigorates his lectures.

Further evidence for fatty acid partitioning among several pathways came from studies led by Angela Wendel, a thoughtful postdoctoral fellow who joined us after completing studies of adipocytes at Ohio State and now prepares students for careers in biology. Using hepatocytes from GPAT1^−/−^ and GPAT4^−/−^ mice, she showed that the two GPAT isoforms can discriminate among fatty acids based on their site of origin: GPAT1, but not the ER-localized GPAT4, is required to incorporate *de novo* synthesized fatty acids into TAG and divert them from oxidation (38). Supporting the concept of differential fatty acid use, graduate student Dan Cooper, whose powers of analytical reasoning have brought him success in cancer biology research, demonstrated that GPAT4 in brown adipocytes normally captures exogenous fatty acids for TAG synthesis, thereby limiting their oxidation and preventing the development of a hypermetabolic state and weight loss (39).

In both liver and adipose, two key transcription factors, sterol regulatory element-binding protein-1 (SREBP1), which is regulated by insulin, and carbohydrate-responsive element-binding protein (ChREBP), which is regulated by carbohydrate availability, upregulate the mRNA for both *de novo* fatty acid synthesis and for GPAT1. Thus, with a high carbohydrate diet and insulin, GPAT1 in hepatocytes captures newly synthesized fatty acids and directs them toward TAG synthesis on the ER. This capture prevents the newly synthesized fatty acids from entering the mitochondria and becoming oxidized—the futile cycle that occurs in the GPAT1^−/−^ mice. There, in the absence of GPAT1, the increased oxidation of fatty acids prevented hepatic steatosis.

In a collaboration with Gerry Shulman’s group at Yale, we fed mice a high-fat diet for 3 weeks. Control mice developed insulin resistance and hepatic steatosis, but again, the GPAT1^−/−^ mice were protected (40). Cynthia Nagle, a highly productive graduate student who now manages a team involved in vaccine studies, led a follow-up collaboration with both the Shulman group and with Chris Newgard’s laboratory at Duke to explore the opposite scenario—adenoviral-mediated overexpression of GPAT1 in the livers of normal mice. Remarkably, within a single week, these mice developed hepatic steatosis and hepatic insulin resistance despite the absence of either obesity or a lipogenic diet (41). This study further highlighted the powerful impact of GPAT1 in driving TAG synthesis and metabolic dysfunction.

In another fruitful collaboration with the Shulman group, Angela Wendel asked whether the absence of GPAT1 would protect genetically obese mice from insulin resistance. Because GPAT1 is required for the SREBP1-mediated induction of hepatic steatosis in *ob/ob* mice, Angie bred mice that lacked the genes that encoded leptin and GPAT1. The absence of GPAT1 reduced hepatic steatosis in the *ob/ob*-GPAT1^−/−^ mice but did not improve the generalized obesity or the animals’ insulin resistance (42). Despite the diminished hepatic TAG and diacylglycerol in the double knockouts, hepatic protein kinase C-ε activation remained elevated, and insulin-stimulated Akt activation remained blunted. Taken together, these data suggest that inactivating GPAT1 might be useful for the treatment of fatty liver but perhaps not diabetes.

Additional studies of the GPAT1^−/−^ mice suggested that its activity contributes to fields remote from hepatic lipid metabolism. Linda Hammond discovered that livers from the GPAT1^−/−^ mice respond to oxidative stress with increases in both apoptosis and proliferation (43). Tal Lewin found that, when fed a lipogenic diet, GPAT1^−/−^ mice accumulate significantly less TAG in their hearts (44). Jessie Ellis and David Paul reported that GPAT1^−/−^ mice are partially protected from developing hepatic tumors induced by diethylnitrosamine (45). Jessie was a dynamic and energetic graduate student who is currently a professor at Wake Forest University studying fatty acid metabolism in brain and muscle, and Dave Paul was a serious postdoc whose current research at UNC focuses on platelet signaling as it relates to thrombosis and hemostasis. A collaboration with Melinda Beck’s group showed that compared with control mice, GPAT1^−/−^ mice infected with Coxsackievirus B3 had increased mortality, higher viral titers, elevated proinflammatory cytokines, and dysregulated splenic dendritic cell function, indicating impairments in both innate and adaptive immune responses (46). Although GPAT1 is most prominently expressed in adipocytes and hepatocytes, these findings in mice devoid of GPAT1 suggest that even the low levels of GPAT1 activity in other tissues may be essential for their normal physiological function.

## Lipid droplets become interesting

While we were studying GPAT, hepatic steatosis, and insulin resistance, I continued to see patients. One little girl who came to the Pediatric Gastrointestinal Clinic had a large fatty liver and ichthyosis, the hallmarks of Neutral Lipid Storage Disease (NLSD-I), a rare genetic disorder in which most of the body’s cells contain prominent lipid droplets. Our literature review placed NLSD patients into two distinct groups (47), and later studies by others identified the two genetic defects: patients with muscle weakness and heart failure (NLSD-M) have mutations in adipocyte triglyceride lipase (ATGL/PNPLA2), and patients with scaley, ichthyotic skin (NLSD-I) have defects in the protein CGI-58, an activator of ATGL.

Because NSLD-I is associated with a number of seemingly unrelated symptoms such as deafness and progressive liver inflammation, it seemed unlikely that CGI-58’s only role was to activate ATGL to release fatty acids from their sequestration in lipid droplets. Ariel Igala—meticulous experimentalist and Kafka devotee from Argentina—showed that although TAG lipolysis is normal in NLSD-I fibroblasts, TAG accumulates in these cells because of defective phospholipid synthesis from the recycling of a TAG intermediate (48). Although we did not understand the underlying biochemistry, this recycling may have occurred because of CGI-58’s second function as a coactivator for the transacylation of acyl-groups from TAG, similar to the transacylation reaction involved in the formation of acyl-ceramides.

**Figure 7:**
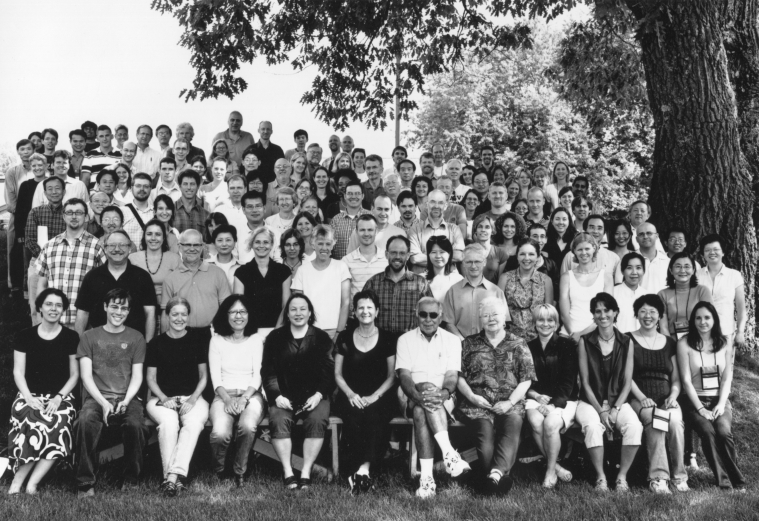
**Attendees at the inaugural FASEB Lipid Droplet Conference in 2007 in Saxtons River, Vermont**. In the first row from the *left* are Patricia Bozza, Derek McMahon, Judy Storch, Hei Suk Sul, Dawn Brasaemle, Rosalind Coleman, Dean Londos, Barbara Corkey, Elizabeth Parks, Debbie Muoio, Lei Li, and Jessie Ellis.

With these studies, we joined a growing number of lipid scientists and cell biologists whose interests spanned a wide range of related areas including the genetic defects underlying the symptoms of NLSD patients, the mechanisms involved in the formation of lipid droplets, and the specific proteins associated with the droplets that regulate TAG lipolysis and synthesis (49). To get this group of scientists together with investigators who were interested in obesity, fatty liver, and diabetes, Dawn Brasaemle (Rutgers University) and I co-founded the FASEB conference on Lipid Droplets in 2007. In Saxtons River, Vermont, we had the opportunity of admiring Bob Farese’s new iPhone, also in its inaugural year, cowering from bats that flew into our rooms, and getting to know a cohort of scientists all tremendously excited to realize the commonality of their interests and the pleasures of interacting and collaborating in a novel field (Fig. 7).

## ACSL, Acyl-CoAs, and lipid channeling

Since the initial studies of diacylglycerol acyltransferase and MGAT, our laboratory focused primarily on acyltransferases, particularly GPAT, but we gradually became interested in an earlier step in complex lipid synthesis: the activation of long-chain fatty acids to form acyl-CoAs. Although at first pursued independently, experiments on these two consecutive steps further enhanced the concept of the intracellular partitioning of lipid metabolites.

Initially, it had been difficult to accept the idea of metabolic channeling for acyl-CoAs because these metabolites are amphipathic and water-soluble. Such properties should allow acyl-CoAs to travel unimpeded through the cytosol or to interact with cellular membranes and with lipid binding proteins. Our experimental results strongly supported the conclusion that cytosolic acyl-CoAs exist in nonmixing pools that partition into differing specific downstream pathways, and we speculated that each one of the different ACSLs might channel its own activated fatty acids into a specific downstream functional pathway. This idea, though simplistic, proved to be conceptually correct.

Evidence for functionally independent pools of acyl-CoA initially came from Ariel Igal’s studies of precursor incorporation of labeled fatty acid and glycerol into TAG and phospholipids in the presence of the acyl-CoA synthetase inhibitor, triacsin C (50). What particularly struck him was the fact that the acyl-CoA synthetase inhibitor, triacsin C, blocked TAG resynthesis while allowing phospholipid synthesis to continue. A mechanistic explanation for this puzzling finding came from the discovery by Tokuo Yamamoto’s group at Tohoku University of five independent but closely related long-chain acyl-CoA synthetases. Studies by Ji-Hyeon Kim, a hard-working graduate student from Korea, showed that the individual acyl-CoA synthetases differed in their sensitivity to inhibition by triacsin C and by thiazolidinedione (51). She and Tal Lewin also confirmed the presence of the different ACSL isoforms on specific membranes within hepatocytes (52, 53), and the fact that in hepatocytes, triacsin C differentially inhibited the incorporation of [^14^C]oleate or [^3^H]glycerol into different lipid end-products, suggesting that each ACSL isoform might be functionally linked to one or more specific metabolic pathways (54).

Supporting the distinct properties of the five ACSLs, Lei O. Li and Matias Caviglia, terrific students from China and Argentina, respectively, found that of the five ACSLs, only ACSL5 was able to complement *E. coli fadD* (55). Lei has since flourished as a director of global clinical studies at a pharmaceutical company in California, and Matias is a professor at Brooklyn College. Working with Matias, postdoctoral fellow Cynthia Van Horn investigated the dissimilar properties of ACSL3 and ACSL6 and identified the latter’s variant form (56).

The literature was particularly confusing at this time because the five long-chain acyl-CoA synthetases and the genes that encoded them were referred to by at least six different names as well as by conflicting numbers. Thus, with the advice of the official human and mouse genome nomenclature committees, I invited all the acyl-CoA synthetase aficionados at the 2003 Lipid Gordon Conference to agree to use a single nomenclature: ACSL1, 3, 4, 5, and 6 (57).

Our investigations into acyl-CoA metabolism had become so compelling that I proposed launching a separate study focused specifically on this topic. However, the NIH Study Section triaged my proposal—likely because it was seen as too speculative and perhaps because it challenged conventional views of metabolic pathways. Fortunately, our local pharmaceutical company Glaxo Wellcome (later GlaxoSmithKline) provided 2 years of funding which allowed us to generate more persuasive evidence supporting the idea that individual ACSL isoforms drive substrate channeling. Then, with NIH funding, we decided to generate a knockout mouse to test the idea that each of the ACSL isoforms had a different metabolic function.

Selecting the ACSL isoform that would be most informative to eliminate engendered considerable laboratory discussion. ACSL4 was interesting because of its preference for polyunsaturated fatty acids, but ACSL1 seemed to be critical for the synthesis of TAG. We knew that ACSL1 is the predominant form in adipocytes (12), that ACSL specific activity increases 100-fold during the differentiation of 3T3-L1 adipocytes (4), and that the amount of its mRNA responds dynamically to dietary changes (58). Concerned, however, that ACSL1 might serve distinct functions in different organs, we decided to create tissue-specific knockouts in those tissues where ACSL1 is most abundantly expressed—liver, heart, skeletal muscle, and adipose tissue (58).

It is extremely disappointing to make a knockout that has no phenotype, and we were all dismayed when Lei Li found negligible changes in the hepatocyte-specific ACSL1^−/−^ mice (59). These mice had only a 20% decrease in the incorporation of [^14^C]oleate into TAG and minimal changes in fatty acid oxidation. Furthermore, they were not protected from diet-induced hepatic steatosis or from insulin resistance. It appeared that the presence of other ACSL isoforms, which comprise at least 50 percent of total ACSL activity in the liver, were playing more prominent roles in these lipid pathways.

The results were more exciting, however, in our adipocyte-specific ACSL1^−/−^ mice. We had predicted that mouse adipocytes lacking ACSL1 would contain little, if any, TAG, and having lost 80% of adipose ACSL activity, would be lipodystrophic. Despite this deficit, and contrary to our prediction of lipodystrophy, the ACSL1^−/−^ mice were heavier than their control littermates and gained weight normally when fed a high fat diet (6). Instead of promoting the synthesis of TAG as we had predicted, adipocyte ACSL1 catalyzes the synthesis of acyl-CoAs that are captured by carnitine palmitoyltransferase-1 for mitochondrial oxidation. This becomes a major problem for mice during the physiological stress of acute cold exposure. Thus, these mice were unable to maintain a normal body temperature when they were kept at 4 ^o^C, despite the upregulation of the genes that are required for energy-uncoupled heat production in brown adipose tissue. What was truly amazing, however, was that despite the absence of ACSL1, the content of acyl-CoA in brown adipose tissue was similar in the control and the knockout mice; it was apparent that these excess acyl-CoAs were unavailable for β-oxidation. The inability of the other ACSL isoenzymes to direct their acyl-CoA products into the β-oxidation pathway, indicated that ACSL1 has a distinct function in brown adipose tissue: its acyl-CoA product is destined for oxidation, and other ACSL isoenzymes cannot compensate for its absence.

Studies of heart-specific ACSL1^−/−^ mice confirmed the importance of ACSL1 for mitochondrial fatty acid oxidation. Since ACSL1 contributes 90% of the total ACSL activity in the heart, its absence forces the heart to rely heavily on glucose to meet its energy demands. Jessie Ellis found that heart-specific ACSL1^−/−^ mice develop cardiac hypertrophy when the heart is almost solely dependent on glucose for contractile energy (60). David Paul, an insightful postdoc, concluded that this pathologic hypertrophy was driven by glucose-mediated activation of the mechanistic target of rapamycin (mTOR) pathway and could be reversed by rapamycin treatment (61). Graduate student Trisha Grevengoed, now happily studying lipids and metabolic dysfunction as a faculty member in Copenhagen, further demonstrated that upregulated mTOR was also responsible for increased autophagy and alterations in mitochondrial structure (62). These results were obtained in mice that lacked cardiac ACSL1 from birth. To examine the transition from normal to pathologic function in adult mice, Florencia Pascual studied a temporal model 2 weeks after a partial knockdown of ACSL1 in the heart. In these mice, Florencia, a native of Uruguay whose command of written English outshone most of the native speakers in our lab, confirmed the development of hypertrophy and the induction of novel mTOR targeted genes that occurs at the onset of substrate switching from fatty acids to glucose, unrelated to abnormalities in diastolic dysfunction (63).

In a collaboration with Robert (Bob) Murphy’s group at the University of Colorado, Trisha Grevengoed discovered that the lack of ACSL1 reduces the content of tetralinoleoyl-cardiolipin, the major species of cardiolipin in heart mitochondria, by 83% (64). Thus, it appears that in addition to handing off acyl-CoAs for mitochondrial oxidation, ACSL1 also determines the acyl-chain composition of heart cardiolipin. Interestingly, however, when Trisha restored normal levels of tetralinoleoyl-cardiolipin, cardiac function did not improve, suggesting that lack of this cardiolipin species is not the cause of the abnormal heart function.

Similar to the specific requirement for ACSL1 in brown adipocytes and cardiac muscle, Lei Li found that exercising skeletal muscle also requires ACSL1. The muscle-specific ACSL1^−/−^ mice ran only half as far as control mice despite having a muscle content of long-chain acyl-CoA that was twice as high. Again, this result confirmed that acyl-CoAs synthesized by other ACSL isoforms are unavailable for β-oxidation (65). As was true in the heart-specific ACSL1^−/−^ mice, the elevated pool of acyl-CoA which had been synthesized by other ACSL isoforms, was unavailable for mitochondrial energy production. Thus, despite their amphipathic nature, those acyl-CoAs synthesized by ACSL1 and those synthesized by other long-chain acyl-CoA synthetase isoforms were indeed located in separate and distinct pools within cells.

How, then, are acyl-CoAs sequestered within the cytosol so that they are available to some downstream pathways and not others? In the liver, ACSL1 has been localized to both the ER and the outer mitochondrial membrane, so we decided to use the unbiased protein interaction discovery technique BioID to find protein interactors. Pamela Young, an astute postdoc from Ireland, targeted ACSL1 constructs to either the ER or to the outer mitochondrial membrane of Hepa1-6 mouse hepatoma cells. She identified multiple protein interactions that differed for each of the two targeted proteins, thereby suggesting that activated fatty acids might move into several specific pathways involved in organelle and lipid droplet interactions, peroxisomal metabolism, and ceramide synthesis (66). Although none of the interacting proteins included carnitine palmitoyltransferase-1 or enzymes in the β-oxidation pathway, Pamela used cultured cells that did not mimic physiological conditions such as fasting or refeeding which might have altered the presence of possible interacting proteins. In fact, recent work by Alan Saltiel’s group at UC San Diego shows that during fasting, ACSL1 in the liver moves from the ER to the mitochondrial membrane in association with TANK-binding kinase-1 (67), consistent with its role in activating fatty acids that are destined for β-oxidation. Postdoctoral fellow Jennifer Frahm’s expertise in mass spectrometry enabled her to complete an extensive study of phosphorylation and acetylation sites on ACSL1; these suggested the presence of numerous potential regulatory and targeting nodes (68).

## Signaling lipid metabolites are also channeled

The concept of metabolic channeling *via* glycerolipid synthetic enzymes was further supported by a third line of investigation that focused on lipid intermediates. Ever since Yasutomi Nishizuka identified *sn*-1,2-diacylglycerol as an activator of protein kinase C, investigators have speculated that other lipid intermediates might also act as signaling molecules. Because TAG accumulation in tissues is strongly associated with insulin resistance, we wondered whether the products of GPAT1, lysophosphatidic acid, and phosphatidic acid, might function as metabolic signals. This idea was compelling because the mitochondrial GPAT1 activity and thus the synthesis of these lipid intermediates would be elevated under dietary conditions that promote insulin resistance.

Postdoctoral fellow Cliona Stapleton brought her strong background in hormone receptor signaling to bear on this problem. Her studies showed that CHO cells constructed to overexpress GPAT1 increased their content of lysophosphatidic acid 6-fold, activated peroxisome proliferator-activated receptor gamma (PPAR-γ), and overexpressed the PPAR-γ target CD36 (69). These features were attenuated after she overexpressed diacylglycerol kinase which diminished endogenous lysophosphatidic acid. The resulting marked inhibition of PPARγ activity indicated that phosphatidic acid might also be a potent inhibitor of PPARγ.

This was an exciting time in our laboratory, and we had fascinating discussions led by Chongben Zhang, a meticulous investigator who was especially interested in lipids as precursors of signaling molecules. Chongben, now a research specialist at UNC, found that overexpressed GPAT1 in primary mouse hepatocytes impairs insulin signaling, inhibits mTORC2 activity, and results in a diminished ability of insulin to suppress glucose production (70). The opposite effect occurred in hepatocytes from GPAT1^−/−^ mice: insulin inhibition appeared to result from a block of mTORC2 activity by phosphatidic acid, especially species that contained palmitate, GPAT1’s preferred substrate and the major fatty acid produced when high SREBP1 upregulates fatty acid synthesis. Extending these studies with our University of Virginia colleague Thurl Harris, Chongben then asked whether other GPAT isoforms would act similarly. Indeed, overexpression of the ER-resident GPAT4 inhibits rictor’s association with the mTOR and mTORC2 activity, and its absence protects against diet-induced insulin resistance. In contrast, overexpression of GPAT3 has no effect (71). Thus, these data indicated that lipid signals derived *via* GPAT1 and GPAT4, likely di16:0-PA rather than diacylglycerol (72), impair insulin signaling in mouse liver and contribute to hepatic insulin resistance. Not only did these data cement links between nutrient excess, TAG synthesis, and hepatic insulin resistance, but they also suggested that GPAT products, like those of the ACSLs, were not equivalent and that a mechanism must exist to partition these intermediates, depending on their enzymatic synthesis.

## Final thoughts and acknowledgments

Although I did not have a long-term plan when I began my research, my goal was to learn as much as possible about enzymes and enzyme regulation critical to the synthesis of TAG. Beginning with enzyme assays and the measurement of initial rates, my laboratory moved to investigate the dynamic changes that occur as fibroblasts differentiate into adipocytes and as neonatal animals alter their energy intake from a prenatal diet of glucose to their postnatal high-fat milk diet. After identifying changes in enzyme activity that rely on membrane interactions, we studied the effects of enhanced and absent expression of GPAT and ACSL isoforms on lipid metabolism and on signaling pathways in cells. Finally, we used physiological interventions on knockout mice, including cold exposure, fasting and refeeding, dietary alterations, and intense exercise to elucidate the functions of specific enzyme isoforms.

Taken together, our studies led to the conclusion that the pathways of complex lipid synthesis and degradation are highly compartmentalized. Both cytosolic and membrane-associated enzymes in lipid-synthetic pathways operate in a concerted manner, as though they were housed within distinct organelles. The metabolism of acyl-CoAs synthesized by the ACSL family, of lysophosphatidic acid and phosphatidic acid synthesized by the GPAT family, and the signaling pathways whose signals are initiated by specific GPAT isoforms, all appear to be functionally separated. Our data strongly support the concept of what Paul Srere and Judit Ovadi defined as metabolons, supramolecular complexes of sequentially interacting metabolic enzymes, proteins, and cellular structural elements that guide metabolites into specific downstream pathways and metabolic fates, depending on physiological circumstances (73). To fully understand these metabolic pathways and their control, future studies should search for the mechanisms that facilitate these collaborative interactions.

**Figure 8:**
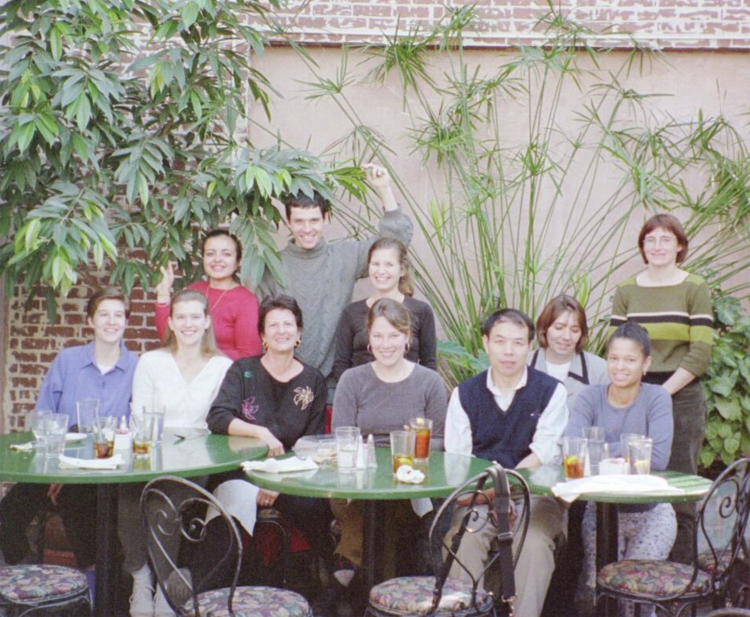
**2001 laboratory lunch**. Seated from the *left*: Tal Lewin, Cynthia Van Horn, RAC, Nicole Schwerbrock, Shuli Wang, Pat Gallagher, Linda Hammond. Standing: Maie El-Sourady, Matias Caviglia, Maria Gonzalez-Baro, and Debbie Granger.

**Figure 9:**
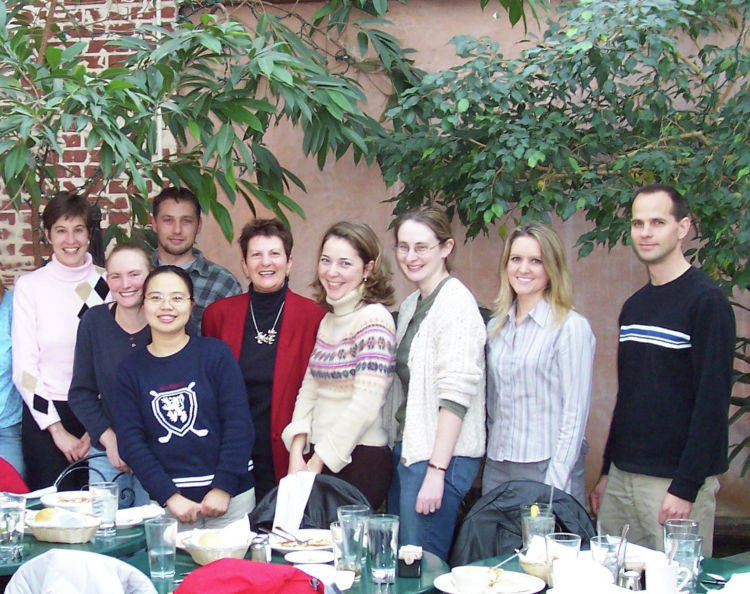
**2005 laboratory lunch**. From the *left*: Tal Lewin, Cynthia Nagle, Hendrik DeJong, Nan Gong, RAC, Diana Mehedint, Cliona Stapleton, Lori Stinnet, and Doug Mashek.

**Figure 10:**
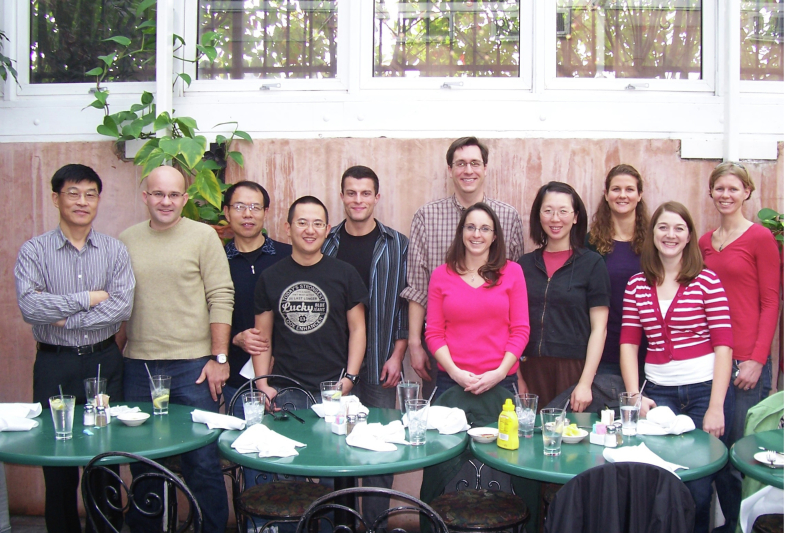
**2009 laboratory lunch**. From the *left*: Chongben Zhang, Matthew Keogh, Shuli Wang, Jucheng Gong, Michael DePetrillo, Eric Klett, Jessie Ellis, Lei Li, Jennifer Frahm, Trisha Grevengoed, and Angela Wendel.

**Figure 11:**
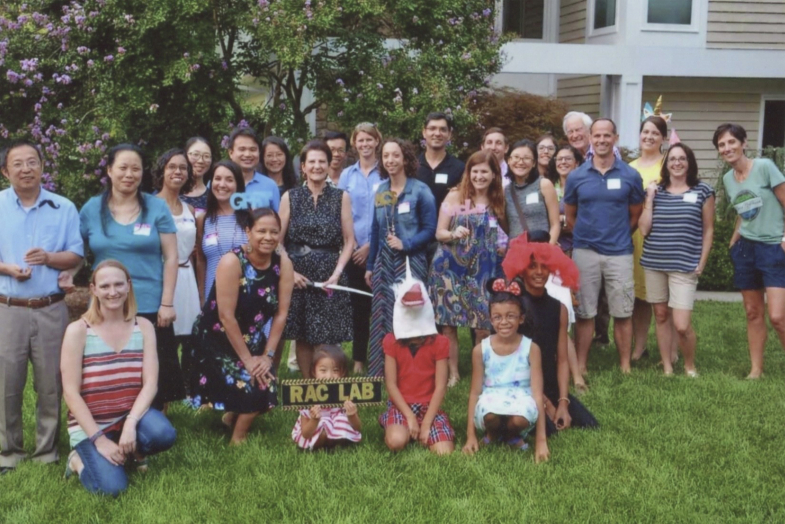
**Retirement party 2019**. First row from *left:* Cynthia Nagle, Linda Hammond. Back row from *left*: Junjiang Sun; Shufen Chen; Michel Alves-Bezzera, Lei Li, Yoseli Quiroga, Eric Yen, Mei-I Yen, RAC, Douglas Lee, Angela Wendel, Theresa D’Aquila, David Paul, Maria Gonzales-Baro, Dan Cooper, Guo Hu, Natalia Rebolledo; Florencia Pascual, James Rolleston, Doug Mashek, Amanda Suchanek, Jessie Ellis, and Debbie Muoio.

Throughout this essay, I have mentioned only some of the individuals who led major projects in my laboratory. I regret that I cannot mention everyone who contributed. Many laboratory members played key roles in other studies that I have not described, and most projects were deeply collaborative. As a group, laboratory members were essential in designing and executing experiments, especially those involving mouse models, and contributed to insightful and productive discussions and journal clubs. I am very proud that I had the opportunity to train truly wonderful students and postdoctoral fellows, the majority of whom were women (Figure 8, Figure 9, Figure 10, Figure 11). I am also proud that seven of my doctoral students won prestigious national prizes, and that eight of my postdoctoral fellows were awarded individual NIH or American Heart Association fellowships. Our laboratory also benefited greatly from the work of numerous enthusiastic undergraduates—about sixty percent of whom went on to careers as physicians and scientists. I like to believe that our laboratory exemplified a strongly communal scientific environment, genuinely committed to the shared success and development of everyone involved.

## Conflict of interest

The authors declare that they have no conflicts of interest with the contents of this article.
